# Effect of Yb on Microstructure and Mechanical Properties of Al-Cu-Mn Heat-Resistant Aluminum Alloys

**DOI:** 10.3390/ma18050958

**Published:** 2025-02-21

**Authors:** Chifu Huang, Hailong Yang, Yu Xiong, Nannan Jia, Junhong Nong, Liwen Pan

**Affiliations:** 1 School of Resources, Environment and Materials, Guangxi University, Nanning 530004, China; hcf1094099919@163.com (C.H.); 18743203440@163.com (H.Y.); xiongyxy@163.com (Y.X.); 18330965585@163.com (N.J.); gxuspark2002@163.com (J.N.); 2 State Key Laboratory of Featured Metal Materials and Life-Cycle Safety for Composite Structures, Guangxi University, Nanning 530004, China; 3 Key Laboratory of High Performance Structural Materials and Thermo-Surface Processing, Education Department of Guangxi Zhuang Autonomous Region, Guangxi University, Nanning 530004, China

**Keywords:** heat-resistant aluminum alloy, Yb, microstructure, mechanical properties, thermal stability

## Abstract

The effect of Yb on the microstructure and tensile properties at room temperature and high temperature (350 °C) of as-cast and T6 heat-treated Al-6Cu-0.4Mn alloys was investigated. The results show that Yb can refine the α-Al primary grain and α-Al + Al_2_Cu eutectic structure. The eutectic structure of the alloy containing 0.3 wt.%Yb has the best refining effect. After the T6 heat treatment, most of the network α-Al + Al_2_Cu eutectic structure disappeared, and many dispersed (200–400 nm) θ′-Al_2_Cu phases were precipitated in the matrix. With the addition of Yb, the amount of θ′-Al_2_Cu phases increases, and the average size decreases from 260 nm in the base alloy to 176 nm in the Yb-containing alloy, which indicates that Yb can promote the precipitation and refinement of the θ′-Al_2_Cu phase; after adding Yb, the high-temperature ultimate tensile strengths (UTS) of the as-cast and heat-treated alloy significantly increase. Both reached their highest when the Yb additions were 0.3 wt.%, reaching 95.5 MPa and 142.3 MPa, respectively, 13.66% and 17.71% higher than the base alloy. The thermal exposure test at 350 °C shows that Yb can improve the θ′-Al_2_Cu phase’s coarsening resistance. Analysis shows that the improvement of mechanical properties at room temperature is due to solid solution strengthening and grain refinement of Yb. The reason for improving mechanical properties at high temperatures is that adding Yb promotes the precipitation, refinement, and thermal stability of the θ′-Al_2_Cu phase.

## 1. Introduction

Higher requirements have been put forward for lightweight, heat-resistant alloys with the emergence of high-power vehicle engines and hypersonic aircraft. High-power engines require heat-resistant components with a temperature-bearing capacity of 300–400 °C. For example, diesel engines used by large engineering vehicles, tanks, and armored vehicles are currently made of heavy cast iron. The temperature-bearing capacity of hypersonic (Mach 3–4) aircraft skin material should also reach 300–400 °C, which can only be manufactured with expensive titanium alloys today (such as the SR-71 Blackbird reconnaissance aircraft [1]), and the cost performance is low. Heat-resistant aluminum alloy is a kind of aluminum alloy with good mechanical properties under high-temperature environments (150–300 °C); that is, it has the properties of being lightweight, high temperature and high strength, oxidation resistance, creep resistance, thermal fatigue resistance, etc. Due to its high specific strength and low price, it is widely used in automotive, aerospace, military, ships, and other fields [2,3,4,5,6,7].

However, the high-temperature strength and heat fatigue performance of traditional cast heat-resistant aluminum alloy is close to the limit state, which cannot meet the temperature resistance requirements of heat-resistant components of new high-power engines and hypersonic aircraft skins. The main reason is that the strengthening phase in aluminum alloy has insufficient thermal stability at high temperatures, which makes it easy to coarsen or dissolve at high temperatures and lose the strengthening effect. Al-Si heat-resistant aluminum alloy use temperature generally does not exceed 230 °C [8,9,10,11]. When Mg is added to Al-Si alloy, the Mg_2_Si phase is precipitated by aging. However, the room temperature strength of the material is improved, and the thermal stability temperature of the Mg_2_Si phase is about 180 °C, so the heat resistance of casting Al-Mg-Si alloy is low. Its working temperature is generally lower than 185 °C [12]. In Al-Cu or Al-Si-Cu alloys, the metastable θ′-Al_2_Cu phase can be precipitated after solid solution aging, and the thermal stability temperature is about 225 °C. However, when the temperature reaches more than 250 °C, the θ′-Al_2_Cu phase will be sharply coarse and then transformed into a stable θ-Al_2_Cu phase with a non-coherent interface with the matrix. High-temperature mechanical properties decreased significantly. Therefore, the overall high-temperature strength level of Al-Cu or Al-Si-Cu cast aluminum alloys is not high and generally can only work below 225 °C [13]. Improving the stability of the metastable θ′-Al_2_Cu phase at high temperatures is considered a significant challenge in the design of heat-resistant aluminum alloys.

Recent studies have shown that the thermal stability of the metastable θ′-Al_2_Cu phase precipitated after aging can be modified by microalloying, and the high-temperature mechanical properties of Al-Cu alloy can be further improved. The principle of microalloying involves introducing various solute atoms, such as some transition metal elements and some rare earth elements (e.g., Mn, Zr, Sc, Y, etc.). In the process of high-temperature aging, these atoms segregate towards the Al/θ′-Al_2_Cu interface, reduce the interface energy, form a solute barrier, and inhibit the coarsening of the θ′-Al_2_Cu phase. There have been many reports on the segregation of micro-alloyed solute to the interface between the Al matrix and precipitated phase, such as Mn and Zr/Ti [14,15], Si [16], Ag [17], Sn [18], Sc [19,20], Mg [21], etc.

SHIN et al. [22] calculated the segregation and interface energy of 34 elements (Mn, Zr, Y, Sc, etc.) at the θ′-Al_2_Cu interface using first principles, and the results showed that the size and volume of solute atoms and their solubility in the θ′-Al_2_Cu phase were closely related to the solute segregation energy. Analysis by scanning transmission electron microscopy (STEM) and atom probe tomography (APT) shows that the segregation of solute atoms towards the coherent and semi-coherent interface between Al and θ′-Al_2_Cu contributes to the stability of the interface and thus improves its thermal stability. However, there are still few experimental reports in this area. JIANG et al. [23] showed that adding Mn to Al-Cu-Sc-Zr alloy can increase the precipitation amount of the θ′-Al_2_Cu phase and refine the size of the θ′-Al_2_Cu phase. The principal analysis shows that the semi-coherent interface segregation of the Mn to θ′ phase is key in accelerating the hardening kinetics and increasing the peak hardness. The interfacial energy is a nucleation barrier to forming a new thermodynamic phase. The interfacial segregation of Mn on the semi-coherent interface reduces the interfacial energy of the θ′ phase, reducing the energy barrier of θ′ nucleation. It thus reduces the driving force required for θ′ nucleation, promoting the precipitation and refinement of the θ′ phase and enhancing the thermal stability of the phase. LAMB et al. [24] found that Al-Cu-Zr-Sc alloy added with Mn showed better mechanical properties after high-temperature thermal exposure, and the Mn-containing alloy suffered less mechanical properties loss after thermal exposure, indicating that the addition of Mn can improve the overall thermal stability of the alloy. The theoretical analysis shows that Mn atoms with low thermal diffusion rate act as a diffusion barrier for Cu atoms during the aging process, preventing Cu atoms from diffusing to the θ′-Al_2_Cu phase and inhibiting the growth of the θ′-Al_2_Cu phase, thus improving the thermal stability of θ′-Al_2_Cu phase. Shyam et al. [14] confirmed through experiments that microalloying Mn and Zr in Al-Cu alloy can raise the working temperature of the alloy to at least 300 °C. The mechanism is thought to be that θ′-Al_2_Cu precipitated after microalloying has higher thermal stability than unalloyed θ′-Al_2_Cu. The reason is that the congruent or semi-congruent interfacial segregation of the micro-alloyed solute atoms between Al and θ′-Al_2_Cu contributes to the stability of the interface, thereby improving the thermal stability of θ′-Al_2_Cu. POPLAWSKY et al. [15] studied the synergistic effect of Mn and Zr/Ti in Al-Cu alloys. When Mn and Zr were added at the same time, the thermal stability of θ′ was increased to 350 °C, while when Mn or Zr was added alone, the θ′ phase only maintained good thermal stability at 200~300 °C, indicating that the two have synergistic stabilization effects on the θ′ phase. The analysis shows that Mn segregates to the θ′ interface faster than Zr/Ti, so it stabilizes θ′ for a long enough time, creating conditions for Zr/Ti with a slow diffusion rate to segregate to θ′ interface and finally forming θ′/L1_2_-Al_3_ (Zr_x_, Ti_1−x_) eutectoid structure, which is the main reason for the significant improvement of θ′ thermal stability. Overall, the continued segregation and eventual L1_2_ formation reduces the interfacial energy, pins the interface, and inhibits θ′ coursing. FU et al. [25] confirmed that the Mn/Ag ratio significantly affects the anti-coarsening ability of the θ′ phase in Al-Cu-Mn-Ag alloys. A 350 °C thermal exposure test and high-temperature tensile test show that when the total content of Ag and Mn is 0.8 wt.%, the precipitation amount of θ′-Al_2_Cu in the alloy reaches the maximum. The size is the smallest when the Mn/Ag ratio is 1:1. The reason is that the Mn and Ag atoms in the alloy show a better synergistic effect. The interface energy of Mn and Ag is at the lowest level when the co-segregation phenomenon occurs at the θ′/Al interface, thus giving the θ′-Al_2_Cu phase the best anti-coarsening performance. It can be seen that Mn is currently considered to be the most effective element for modifying the thermal stability of the θ′-Al_2_Cu phase, and its co-addition with some other elements also shows a better co-modification effect. However, there are few reports on the co-modification of θ′-Al_2_Cu between Mn and other elements, which is worthy of further study.

The addition of rare earth elements to Al-Cu alloy can form Al-Cu-Re intermetallic compounds with high thermal stability, which can prevent grain boundary slip and dislocation movement at high temperatures and improve the heat resistance of the alloy. AMER S et al. [26] studied the microstructure and mechanical properties of Al-4.4Cu-2.5Yb alloy. The results show that the solid solution containing the Yb matrix, Al_8_Cu_4_Yb phase, and Al_3_Yb phase can be used as inhibitors of recrystallization of the alloy. The Al_8_Cu_4_Yb phase and Al_3_Yb phase are not easily decomposed or coarse at high temperatures. They can effectively nail the grain boundaries and dislocations, inhibit the movement of the grain boundaries and dislocations, improve the microstructure stability of the alloy, and then improve the heat resistance of the alloy. XIAO et al. [27] studied the effect of Yb content on Al-Cu-Mg-Ag alloy and found the addition of 0.10 wt.%~0.35 wt.% Yb could accelerate the age-hardening process of the alloy and increase the maximum hardness and tensile strength from room temperature to 300 °C. In addition, Yb can reduce the size of the precipitated phase, reduce the precipitation temperature of the Ω phase, and improve the density and thermal stability of the Ω phase between 200 and 300 °C. The reason is that the segregation of Yb at the interface of Ω phase can delay the diffusion of Cu and reduce the coarsening rate of Ω phase. ZHANG et al. [28] studied the effect of Yb on the mechanical properties of 2519A alloy at high temperature (300 °C). The results show that the lattice distortion energy and vacancy energy generated by adding Yb atoms are conducive to the nucleation of θ′-Al_2_Cu near Yb atoms, thus increasing the number and density of θ′-Al_2_Cu particles. Meanwhile, the diffusion of Cu atoms is limited by Yb atoms, and the coursing rate of θ′-Al_2_Cu particles is reduced at 300 °C. The reason is that the diameter of the Yb atom (0.194 nm) is more significant than that of the Al atom (0.143 nm), so when it enters the matrix, it will inevitably cause excellent lattice distortion and increase the system’s energy. More supersaturated vacancies are clustered around the Yb atoms to keep the system energy low. Since the θ′ phase is semi-coherent with the Al matrix, it is prone to dislocation and vacancy nucleation, increasing the number of θ′ [29]. More supersaturated vacancies are clustered around the Yb atoms to keep the system energy low.

Considering that the microalloying of Mn and other elements may have a synergistic effect on the thermal stability modification of the θ′-Al_2_Cu phase, the positive effect of rare earth Yb elements on the heat resistance of Al-Cu alloy and the thermal stability modification of θ′-Al_2_Cu by the combination of Yb and Mn have not been reported. Based on Al-Cu-Mn alloy, the effects of rare earth Yb on the microstructure and mechanical properties at room temperature and high temperature of Al-Cu-Mn alloy were studied. The mechanism of Yb influence on the coarsening resistance of the θ′-Al_2_Cu phase was investigated by thermal exposure experiments, aiming at exploring the feasibility of ytterbium microalloying to improve the thermal stability of heat-resistant aluminum–copper alloy θ′-Al_2_Cu enhanced phase.

## 2. Materials and Methods

The raw materials used for melting alloys in this experiment are industrial pure aluminum block (99.9%), Al-50Cu, Al-10Mn, and Al-10Yb master alloys, and the melting crucible is a high-purity graphite crucible. In addition, the other agents used were a melt covering agent (50% KCl + 50% NaCl powder), a refining agent (CCl_6_ powder), and graphite crucible coating (Na_2_SiO_3_:ZnO = 1:3). Table 1 shows the nominal composition of the experimentally prepared alloys. These reagents are from Xiongrun Chemical, Nanning, China.

**Figure 1:**
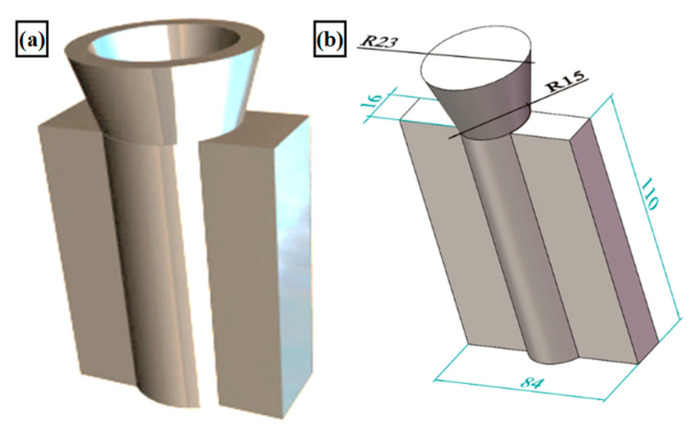
Shapes and dimensions of casting molds and ingots: (**a**) steel mold; (**b**) ingot casting.

The alloy was melting on a graphite crucible in a medium-frequency induction furnace (Model: M.M.F. 00008, Yihui Castino, Guangzhou, China). In the first step, the inner wall of the graphite crucible was uniformly coated with the matched coating (Na_2_SiO_3_:ZnO = 1:3, water mixed with coating 1:1), which was dried in a muffle furnace at 250 °C for 30 min. In the second step, the dried pure aluminum block was placed into the graphite crucible and heated by electricity. After the ingot had completely melted, the temperature of the aluminum liquid was measured using a K-type thermocouple. The power was adjusted to keep the melt temperature at 760 °C or so, slagging. In the third step, Al-50Cu and Al-10Mn master alloy were added, and in the aluminum liquid surface, were evenly sprinkled with covering agent, thermal insulation 5–6 min after slagging. The fourth step was to reduce the heating power to reduce the melt temperature to about 690 °C by adding Al-10Yb, a covering agent, holding time of 5 min, manual stirring of the melt, and slagging. Step 5 was to increase the heating power to heat the melt to about 740 °C, add hexachloroethane, and manually stir the melt to degas; degas time was 2 min, with two consecutive degas. In the sixth step, the melt was left to stand for 2 min, slagging was performed, and finally, the melt was poured into the metal mold with a preheating temperature of 250 °C (shown in Figure 1), and the casting was solidified and cooled, then de-molded.

The T6 heat treatment of the micro alloyed alloy was carried out using a YYX1200-40 JINDUN (Shiyan electric furnace, Shanghai, China) resistance furnace, in which the process parameters for the Al-6Cu-0.4Mn-xYb heat treatment test were as follows: solid solution process 540 °C/6 h + water quenching at 25 °C, aging process 170 °C/6 h + air-cooling, and the heating rate was 10 °C/min. The optimized high-temperature mechanical properties of the T6 heat-treated state and the base alloy were selected for thermal exposure experiments at 350 °C for different times (12 h and 24 h) at the same time to investigate the effect of microalloying elements on the heat-resistant stability of θ′-Al_2_Cu.

Microstructural observations and analyses of the fracture surfaces of the metallographic and tensile samples were carried out using a Phenom ProX benchtop scanning electron microscope (backscattered electron detector, accelerating voltage of 10 or 15 kV, standard beam current) and an accompanying energy spectrometer (Phenom-World, Eindhoven, the Netherlands). Physical phase analysis was performed using a MiniFlex 600 X-ray diffractometer (Rigaku Corporation, Tokyo, Japan) with a scanning speed of 6°/min and scanning angles from 20° to 80°. Jade 6.5 software was used to determine the type of physical phase in the alloys.

**Figure 2:**
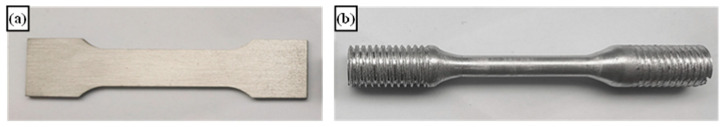
Real pictures of tensile specimens ((**a**): room temperature tensile specimen, (**b**): high-temperature tensile specimen).

The specimen specifications follow the ASTM international standard for tensile testing for metallic material. The room-temperature tensile specimen was a plate (Figure 2a), gauge length 25 mm. The high-temperature tensile specimen was a round bar (Figure 2b), gauge length 30 mm. Room-temperature and high-temperature tensile tests are carried out by a BWN100KN series electronic universal testing machine with a high-temperature heating system (Airma, Shenzhen, China). Before the tensile test, the specimens were pre-stretched, and finally, the tensile test was performed at a rate of 0.5 mm/min. At room temperature, the specimen was pre-stretched at 0.5 mm/min to approximately 200 N before the test was carried out; at high temperature, the specimen was pre-stretched at the same rate to approximately 200 N and then held at 350 °C for 20 min before the test was carried out. After tensile testing, the ultimate tensile strength (UTS, MPa), yield strength (YS, MPa), and fracture strain (FS, %) elongation (EL, %) of the alloys were obtained.

## 3. Results

### 3.1. Microstructure of As-Cast Alloys

**Figure 3:**
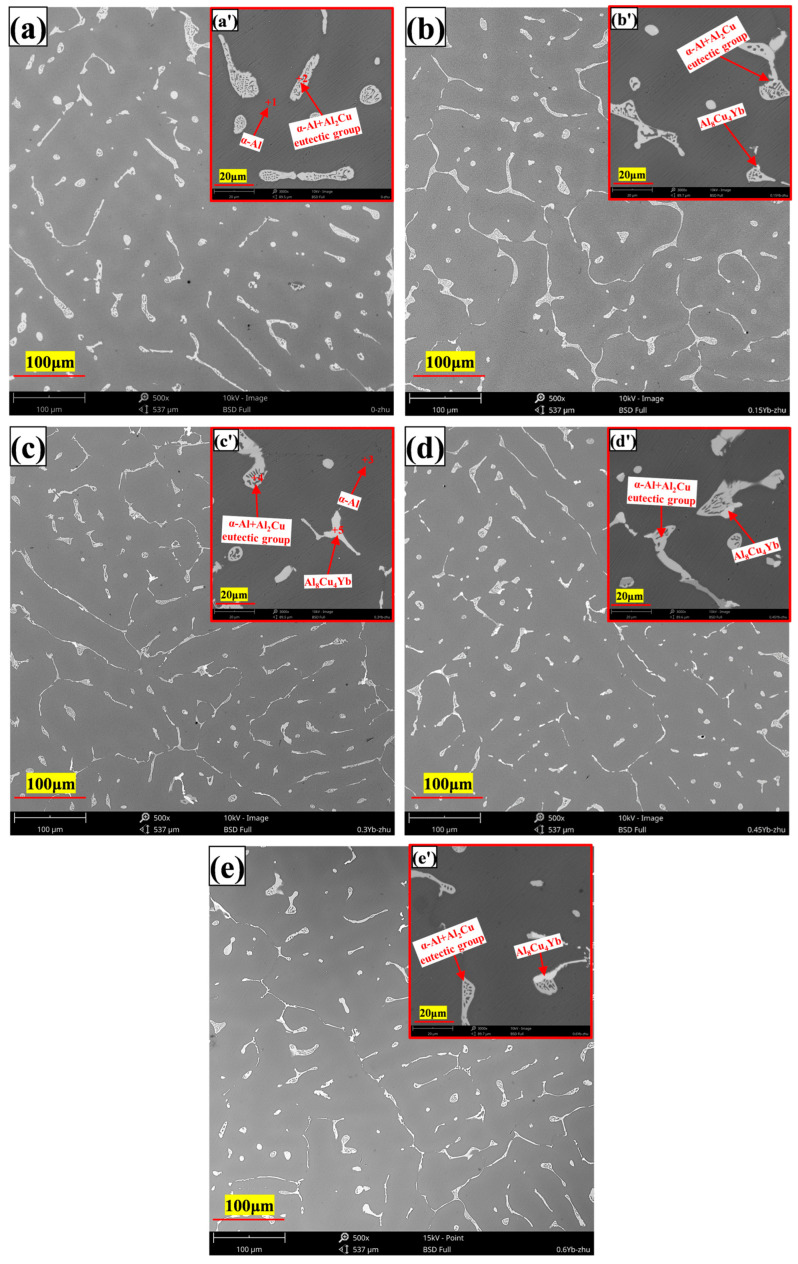
Microstructure of as-cast alloys ((**a**,**a’**) = 0%Yb; (**b**,**b’**) = 0.15% Yb; (**c**,**c’**) = 0.3% Yb; (**d**,**d’**) = 0.45% Yb; (**e**,**e’**) = 0.6% Yb, wt.%).

**Figure 4:**
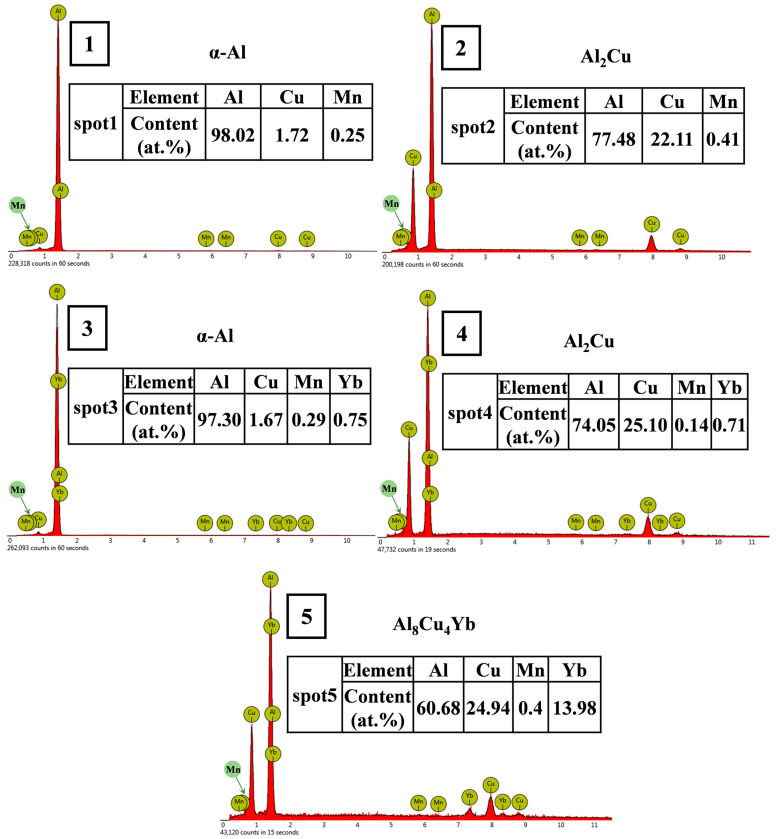
EDS analysis of points 1 to 5 in Figure 3.

**Figure 5:**
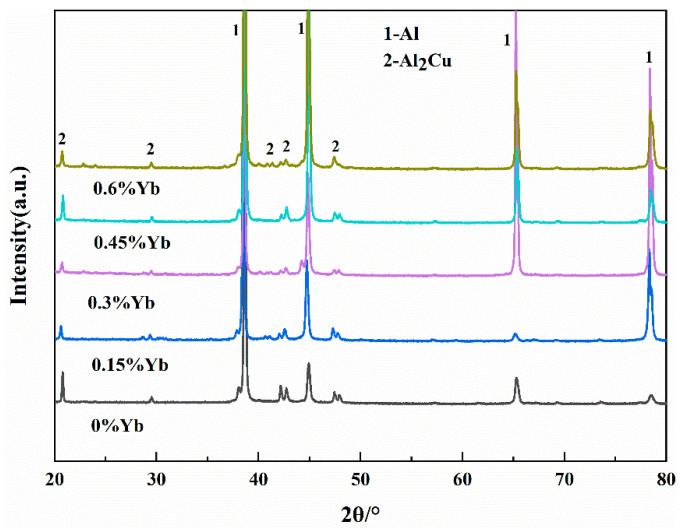
X-ray diffraction pattern of as-cast alloy.

The microstructure of the as-cast alloy is shown in Figure 3, and the EDS microzone composition analysis of each phase is shown in Figure 4. The X-ray diffraction patterns of the alloy are shown in Figure 5. It can be seen from Figure 3 that the as-cast microstructure of the alloy consists of α-Al (spot 1, 3), θ-Al_2_Cu (spot 2, 4), and Al_8_Cu_4_Yb (spot 5) phases. The dark grey α-Al is the matrix phase with a dendritic crystal structure; the light white banded or reticulated phase mixed with the dark grey phase is the α-Al + θ-Al_2_Cu eutectic structure, which is distributed at the grain boundaries or interdendritic regions; and a small number of skeletal bright white Al_8_Cu_4_Yb phase attached to the eutectic structure. According to the Al-Cu binary equilibrium phase diagram, the Cu content between the maximum solid solubility (5.65%) and the Al-Cu eutectic point (33.2%) belongs to the sub-eutectic alloy.

Therefore, the primary phase is α-Al, and the eutectic structure α-Al + Al_2_Cu will form at the grain boundary when the primary α-Al phase finishes its solidification. It can be seen that the addition of the Yb element affects the size of α-Al grains and the eutectic structure. The microstructure photos of the as-cast alloy show that the α-Al grain or dendrite size is refined after adding Yb, and the average grain size decreases from 60 μm of the matrix alloy to 40 μm of the alloy containing Yb. This is consistent with previous research [27,28]. As seen from the θ-Al_2_Cu grain spacing, the α-Al dendrite refinement starts to be insignificant when the Yb content is more than 0.3 wt.%. It can explain the refinement of α-Al dendrite according to the Hume–Rotnery theory [30]. When the difference between the atomic radii of two elements is greater than 15%, the solid solution formed has low solid solubility, and the difference between the atomic radii of Yb and Al is 35.7%, so it can be inferred that the solid solubility of Yb in aluminum is extremely low. ZHANG et al. [31] determined that the solubility of Yb in aluminum is about 0.024 ± 0.004 at.%, and the equilibrium distribution coefficient K0 of Yb is much less than 1. Therefore, during solidification, due to the extremely low solubility of Yb in aluminum, it will be easy to enrich and agglomerate at the front of the solid–liquid interface, preventing Al atoms outside the solidified interface from diffusing into the solidified α-Al phase and, at last, refining the α-Al primary phase.

On the other hand, the enrichment of Yb at the solidification interface during the solidification process also leads to solute redistribution, resulting in the supercooling of the components of the solidification interface and ultimately speeding up the solidification of the residual liquid surrounding the α-Al grains, and indirectly refining the size of α-Al grains [32]. Due to the constitutional supercooling of the residual liquid around the primary α-Al grains, the size of α-Al + Al_2_Cu elongated reticular eutectic structure decreases with the Yb content increasing, which can be seen in Figure 3; 0.3 wt.% Yb alloy has the best effect on eutectic structure refinement. When the Yb content is 0.3 wt.%, the micrometer-sized Al_8_Cu_4_Yb begins to appear. The number of Al_8_Cu_4_Yb phases increases increasingly as the Yb content increases, while the size is coarser with the content Yb increasing. However, since the total number of Al_8_Cu_4_Yb phases is not large, the diffraction peak of this phase is not seen in the diffraction pattern of Figure 5, but based on the EDS analysis in Figure 4 and some references [26,33], we conclude that the phase is Al_8_Cu_4_Yb.

### 3.2. Mechanical Properties of the As-Cast Alloys

**Figure 6:**
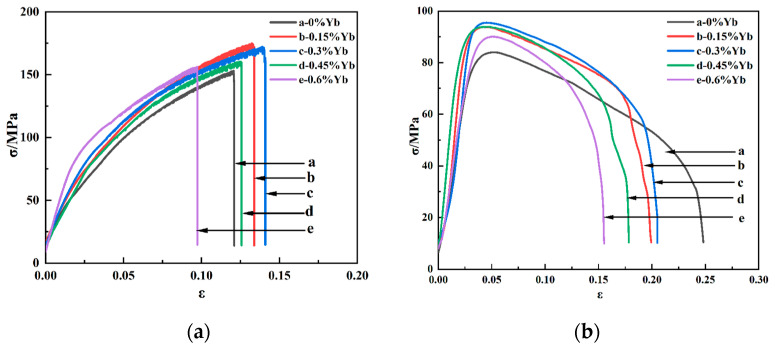
Tensile stress–strain curves for cast alloys: (**a**) room temperature; (**b**) 350 °C.

Figure 6a and Figure 6b show the tensile stress–strain curves of the as-cast alloy at room temperature and high temperature (350 °C), respectively, and the corresponding tensile mechanical properties are shown in Table 2 and Table 3.

**Figure 7:**
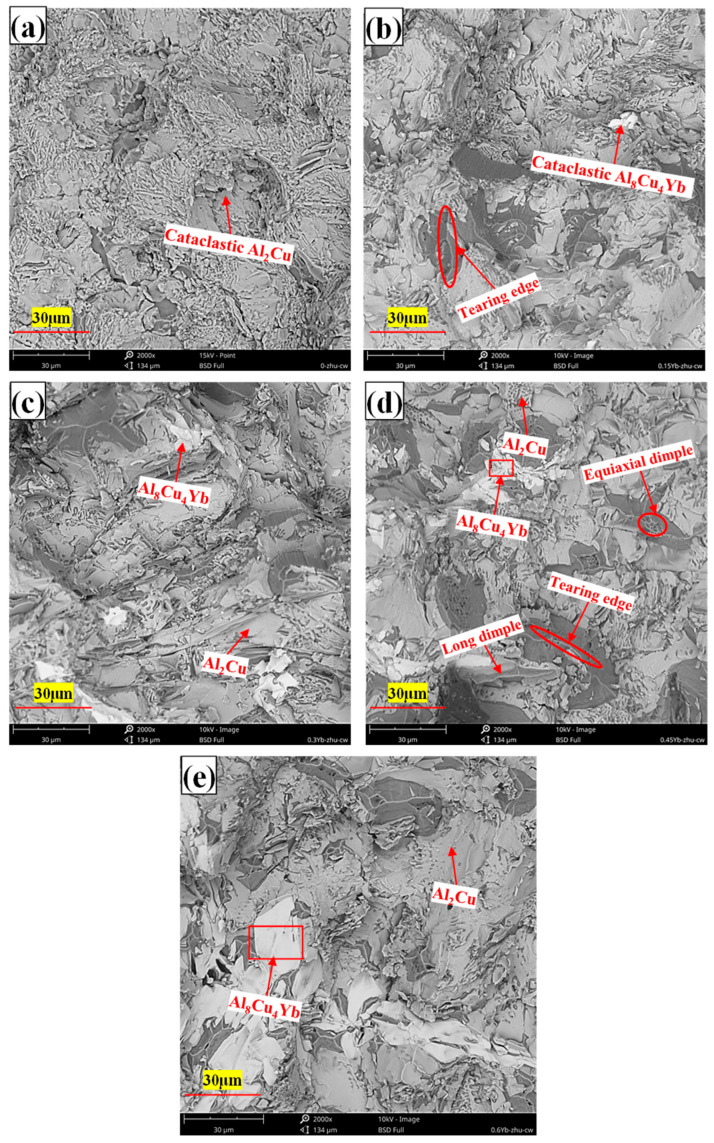
Fracture morphology of cast alloys under room temperature stretching ((**a**) = 0%Yb; (**b**) = 0.15%Yb; (**c**) = 0.3%Yb; (**d**) = 0.45%Yb; (**e**) = 0.6%Yb, wt.%).

As can be seen from Table 2, whether at room temperature or high temperature, the mechanical properties of the alloy after adding Yb are better than those of the base alloy. The alloy’s ultimate tensile strength and yield strength at room and high temperatures increased first and then decreased with Yb content. The mechanical properties of the alloy are the best when the Yb content is 0.3 wt.% and the ultimate tensile strength and yield strength at room temperature reach 174.85 MPa and 113.84 MPa, respectively, 14.21% and 18.34% higher than the base alloy. When the content of Yb exceeds 0.45 wt.%, its mechanical properties show a decreasing trend. The improvement of tensile properties at room temperature after adding Yb is mainly due to solid solution and fine-grain strengthening. The energy spectrum analysis of the matrix shows that the α-Al matrix phase contains a small amount of Yb element (Figure 4); the atomic radius of Yb is 0.194 nm, and the atomic radius of Al is 0.143 nm. The solution of Yb in the matrix generates many dislocation and lattice distortion stress fields. The interaction between the lattice distortion stress field and the dislocation stress field hinders the slip of the dislocation in the matrix, thus enhancing the deformation resistance of the matrix. However, due to the extremely low solubility of Yb in the aluminum matrix at room temperature [34], Yb’s solid solution strengthening effect is very limited. In addition, due to the large concentration of Yb at the solidification interface front, it acts as an inhibitor of α-Al grain growth and refines the α-Al grain. According to the Hall–Petch formula [35], under the same shear stress, refined grains require more significant force than coarse grains to activate the dislocation source of neighboring grains, which is the main reason for improving room temperature mechanical properties. When Yb is 0.3 wt.%, the average grain size of as-cast alloy α-Al is reduced by about 10% compared with the base alloy. A small amount of micron-level Al_8_Cu_4_Yb will hinder the propagation of grain boundary cracks and the occurrence of slip during deformation. However, the coarse and brittle Al_8_Cu_4_Yb will easily split the matrix during the tensile process, resulting in stress concentration and accelerating material fracture. As seen from the fracture morphology in Figure 7d,e, smooth cleavage surfaces are produced in some of the coarse Al_8_Cu_4_Yb phases. Therefore, the tensile mechanical properties decrease significantly when the addition of Yb reaches 0.6 wt.%.

**Figure 8:**
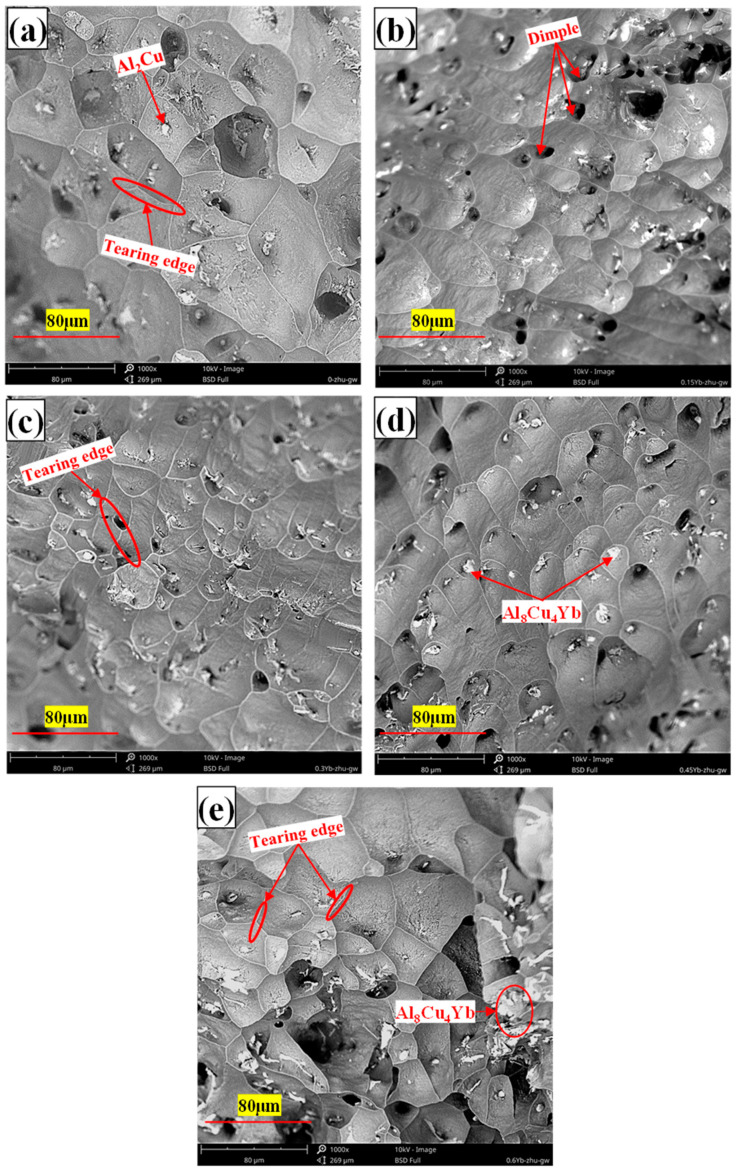
Fracture morphology of as-cast alloy under high-temperature tension ((**a**) = 0%Yb; (**b**) = 0.15%Yb; (**c**) = 0.3%Yb; (**d**) = 0.45%Yb; (**e**) = 0.6%Yb, wt.%).

It can be seen from Table 3 that the addition of Yb also helps to enhance the high-temperature tensile properties of the as-cast alloy. With the increase of Yb addition, the tensile strength of the alloy increases first and then decreases. When the addition of Yb is 0.3 wt.%, the mechanical properties of the alloy are the best, and the ultimate tensile strength and yield strength reach 95.46 MPa and 85.21 MPa, respectively, which are 13.65% and 19.11% higher than that of the base alloy. When the content of Yb exceeds 0.45 wt.%, the mechanical properties begin to decline. The improvement of the high-temperature performance of the as-cast alloy is mainly due to grain refinement and strengthening of the solid solution. When the short-time tensile test is carried out at 350 °C, the temperature is lower than the equal strength temperature of aluminum alloy [36], and the grain boundary strength is greater than the intercrystalline strength. After adding Yb, the alloy matrix grains are refined, the number of grain boundaries is increased, and the grain boundary strengthening effect is significant, so the alloy’s tensile strength and yield strength are improved. Considering that the atomic radius of Yb is more significant than that of Al, the lattice distortion stress field caused by the solid solution of the aluminum matrix also hinders the dislocation movement, which is also the reason for the improvement of high-temperature performance. However, although the Al_8_Cu_4_Yb phase at the grain boundary is a high-temperature stable phase, the coarse and brittle Al_8_Cu_4_Yb phase plays a harmful role in the as-cast state. From the high-temperature tensile fracture morphology, it can be seen that the coarse Al_8_Cu_4_Yb phase often has large cracks and fragmentation in its interior, which is the focus of stress concentration, as seen in Figure 8d,e that large and shallow dimples often surround the coarse Al_8_Cu_4_Yb phase, indicating that the coarse Al_8_Cu_4_Yb phase accelerates the formation of micropores around it, accelerates the connectivity of micropores, and accelerates the fracture of materials. The more Yb is added, the more noticeable this situation is, so the high-temperature mechanical properties of the as-cast alloy decrease in strength and plasticity when the content of Yb is higher, as shown in Table 3.

### 3.3. Microstructure of Al-6Cu-0.4Mn-xYb Alloy After T6 Heat Treatment

**Figure 9:**
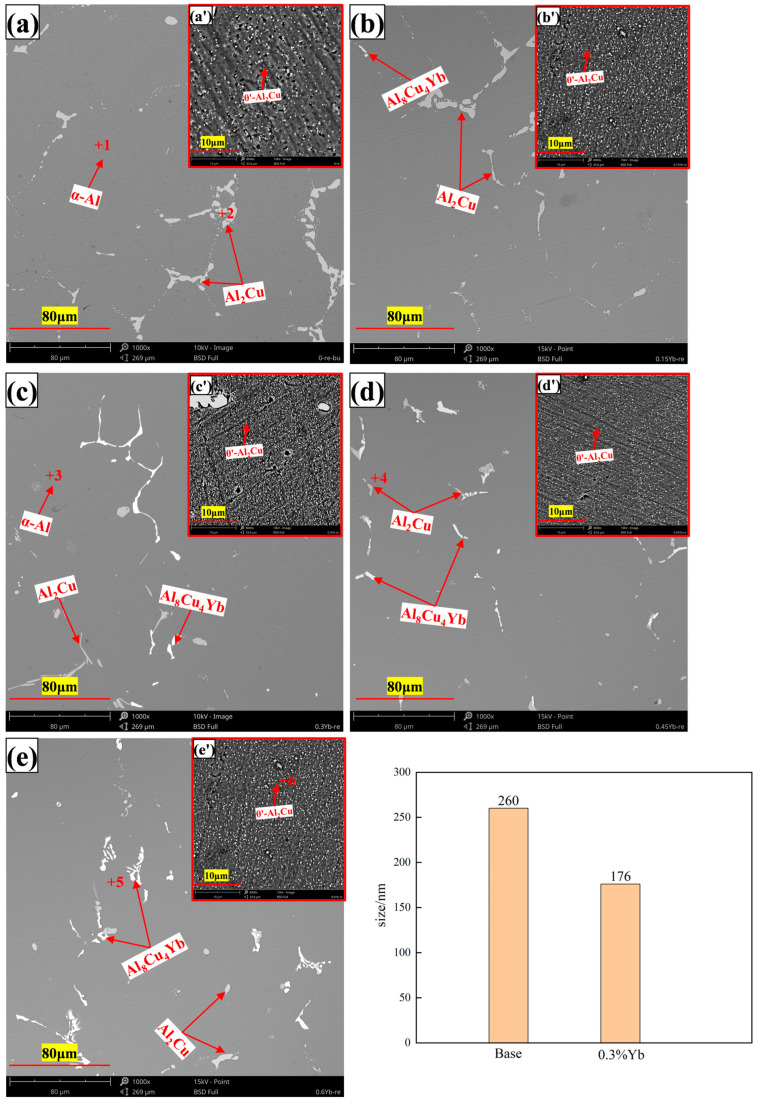
Microstructure and θ′-Al_2_Cu average particle size of T6 heat-treated alloy ((**a**,**a’**) = 0%Yb; (**b**,**b’**) = 0.15%Yb; (**c**,**c’**) = 0.3%Yb; (**d**,**d’**) = 0.45%Yb; (**e**,**e’**) = 0.6%Yb, wt.%).

**Figure 10:**
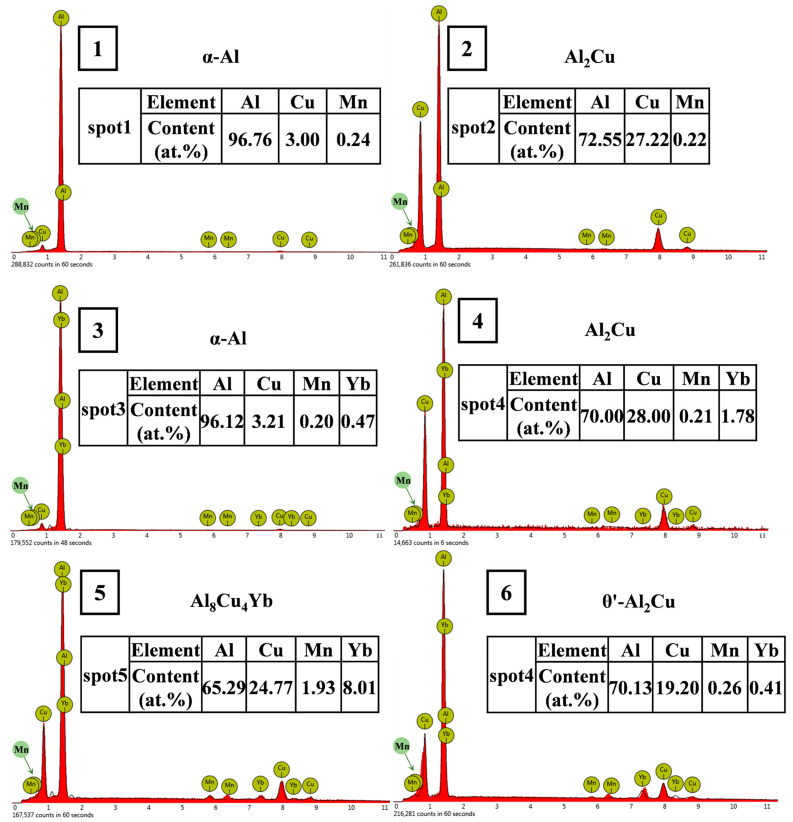
EDS analysis of points 1 to 6 in Figure 9.

**Figure 11:**
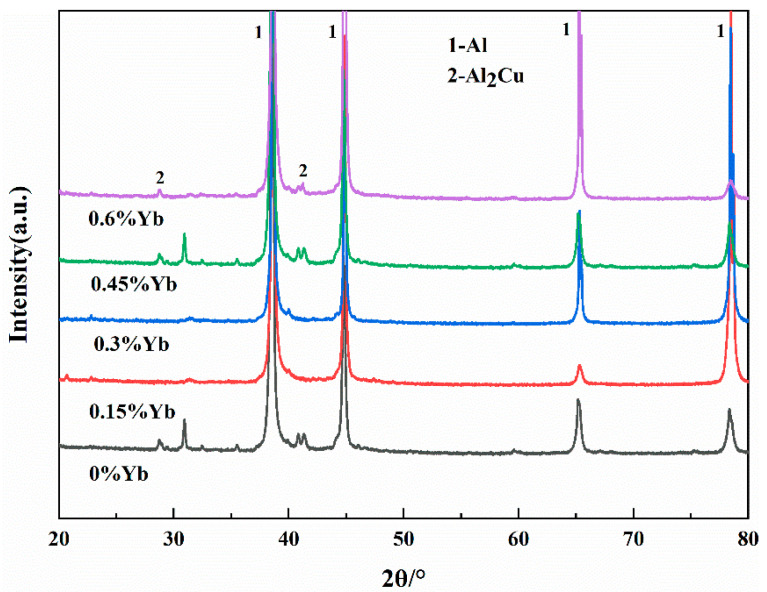
X-ray diffraction pattern of T6 heat-treated alloy.

The microstructure photos of the heat-treated alloy are shown in Figure 9, the low-magnification times photos in Figure 9a–e, and the high-magnification times photos in Figure 9a’–e’. The microcomponent analysis of each point in Figure 9 is shown in the energy spectrum diagram in Figure 10. Figure 11 shows the X-ray diffraction pattern of the alloy. The phase composition of the heat-treated alloy has not changed and is still composed of α-Al (spot 1, 3), θ-Al_2_Cu (spot 2, 4), and Al_8_Cu_4_Yb (spot 5) phases. However, the microscopic morphology changed considerably. The low-magnification photographs show that after heat treatment, many reticulated α-Al + Al_2_Cu eutectic structures at the grain boundaries disappear, and the residual Al_2_Cu phases are clustered into spheres or strips. Due to the strong thermal diffusion during solid solution treatment at 540 °C, most of the coarse Al_2_Cu phase that initially existed in the cast state is dissolved into the aluminum matrix (the limit solid solubility of Cu in the aluminum matrix is 5.65 wt.%). In addition, to achieve thermodynamic stability, the residual Al_2_Cu phase appears spheroidized, and the average size of the spheroidized particles is 5 μm. After aging treatment at 170 °C, the θ′-Al_2_Cu phase (spot 6) precipitates from a supersaturated solid solution; the size of these precipitates decreases first and then increases, mainly when the amount of Yb is increased, the amount of precipitation also increases, the number of particles increases to a certain extent, andthe particle spacing decreases, the trend of particle consolidation and growth. Therefore, the microstructure characteristic of θ′-Al_2_Cu particles refined and then coarsened in Figure 9 is finally formed. The Al_8_Cu_4_Yb phase does not dissolve after T6 heat treatment, indicating that the phase has good thermal stability.

It can be seen from the high-magnification microstructure photos that the small and dispersed θ′-Al_2_Cu phase is precipitated from the matrix, with a size of about 100–300 nm. In general, the size of θ′-Al_2_Cu precipitated from the matrix after adding Yb is smaller and more dispersive than that of the base alloy without adding Yb [27,28]. The average particle size of θ′-Al_2_Cu of the base alloy without Yb and the alloy containing 0.3 wt.%Yb was measured using Nano Measure software 1.2. It was found that θ′-Al_2_Cu precipitated with 0.3 wt.%Yb alloy was 32% finer than the base alloy. It is shown that Yb can significantly promote the precipitation and refinement of the θ′-Al_2_Cu phase. According to the reference [34,37], Yb has a low thermal diffusion coefficient in the aluminum matrix (D_Yb_ = (6 ± 2) × 10^−17^ m^2^/s, 300 °C), while Mn (D_Mn_ = 7.4 × 10^−18^ m^2^/s, 500 °C), Al, and Cu have higher thermal diffusion coefficients than Yb. In the aging process, Yb atoms with slow diffusion speed will block and inhibit the diffusion and movement of Al, Cu, and Mn atoms, forming Al, Cu, and Mn atomic clusters like a “traffic jam”. When the atomic clusters grow to the critical nucleation radius of the θ′-Al_2_Cu phase, the θ′-Al_2_Cu phase begins to nucleate and grow. A large number of Yb atoms in the matrix promotes the increase of the overall quantity and decreases the size of θ′-Al_2_Cu particles. A trace amount of Mn atoms can be detected from the precipitated phase θ′-Al_2_Cu (Figure 10), which indicates that Mn participates in the clusters and promotes the atomic clusters. It has been reported that in 2519A aluminum alloy, with the addition of Yb, the diffusion of Cu may be inhibited by large Yb atoms aggregated in vacancy clusters, and the rate of roughening of the θ′-Al_2_Cu phase is reduced [28]. In addition, Yb atoms form a significant lattice distortion in the aluminum matrix, resulting in many dislocations during quenching. According to the reference [38], these dislocations can become the nucleation sites of θ′-Al_2_Cu during aging, which is also the reason for promoting θ′-Al_2_Cu phase precipitation and refinement.

### 3.4. Tensile Properties of Al-6Cu-0.4Mn-xYb Alloy After T6 Heat Treatment

**Figure 12:**
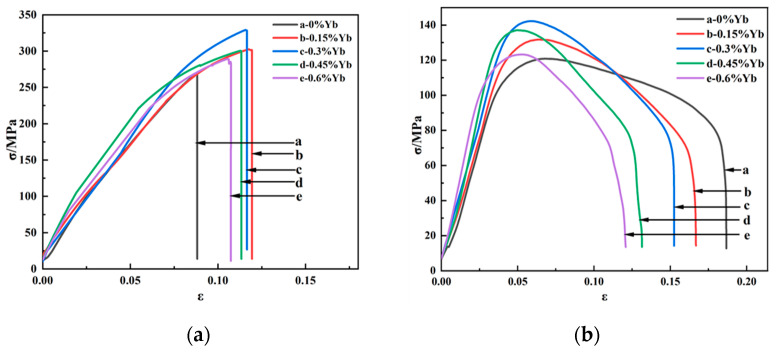
Tensile properties of alloys after heat treatment of T6: (**a**) room temperature; (**b**) 350 °C.

Figure 12 shows the tensile stress–strain curves at room temperature and high temperature (350 °C) after heat treatment of the alloy. Table 4 and Table 5 show the corresponding tensile mechanical properties. As can be seen from Table 4, the tensile strength and yield strength of the heat-treated alloy at room temperature are significantly improved compared with that of the as-cast alloy. The ultimate tensile strength of the heat-treated alloy at room temperature is 154.13 MPa higher than that of the as-cast alloy, an increase of 88%, indicating that the heat treatment plays a vital role in the mechanical properties of the alloy. The tensile strength of the heat-treated alloy at room temperature increases first and then decreases with the increase of Yb content. The ultimate tensile strength and yield strength of the alloy with Yb content of 0.3 wt.% are the highest, reaching 328.98 MPa and 272.86 MPa, respectively, which are 21.63% and 33.95% higher than that of the base alloy, respectively.

Similarly, the heat-treated alloy’s high-temperature tensile mechanical properties are significantly improved compared to that of the cast alloy, and the ultimate tensile strength and yield strength are 49% and 46% higher than that of the as-cast alloy, respectively. The tensile strength and yield strength also increased first and then decreased with the increase of Yb content. When the Yb content was 0.3 wt.%, the ultimate tensile strength reached 142.26 MPa. The tensile strength and yield strength of the alloy with Yb addition is higher than those of the base alloy without Yb addition, indicating that the addition of Yb has a significant effect on the mechanical properties of the alloy at room temperature and high temperature. Table 6 lists the tensile strengths of some heat-resistant aluminum alloys reported in recent years at elevated temperatures. The alloys studied in this paper have better properties, indicating potential for industrial applications. Consider, for example, applications such as compressor blades, impellers, and magazines for aircraft engines and pistons and cylinder heads for automobile engines.

Adding Yb dramatically improves the mechanical properties of the heat-treated alloys at both room and high temperatures. The main reason is the change of microstructure caused by heat treatment and Yb addition. After high-temperature solution treatment and aging, many dispersive and small θ′-Al_2_Cu particles with a size of about 100–300 nm were precipitated from the matrix. According to the literature [22,49], the θ′-Al_2_Cu phase and Al matrix are coherent or semi-coherent, and the interface is firmly combined. Dense and firmly bonded θ′-Al_2_Cu particles will play a role in nailing and preventing the matrix dislocation slip, thus significantly improving the alloy’s tensile strength and yield strength, which is the classic Orowan strengthening mechanism [50,51]. According to the Orowan strengthening mechanism, the more non-deformed second phase strengthened particles, the more uniform the distribution, and the smaller the particle spacing, the more significant the strengthening effect. The number of θ′-Al_2_Cu particles increases first and then decreases with the increase of Yb addition, while the particle size decreases first and then becomes coarse. Therefore, the mechanical properties of the alloy at room temperature and high temperature also increase first and then decrease with the increase of Yb addition, indicating that θ′-Al_2_Cu precipitation after aging plays a decisive role in the mechanical properties of the alloy. Although the matrix contains a small amount of Yb, the solid solution strengthening effect is still limited. Since the solid solubility is minimal and the solid solution content does not change with the additive amount at the same temperature, the increase in the number of θ′-Al_2_Cu particles and the decrease in size are the main reasons for improving mechanical properties.

**Figure 13:**
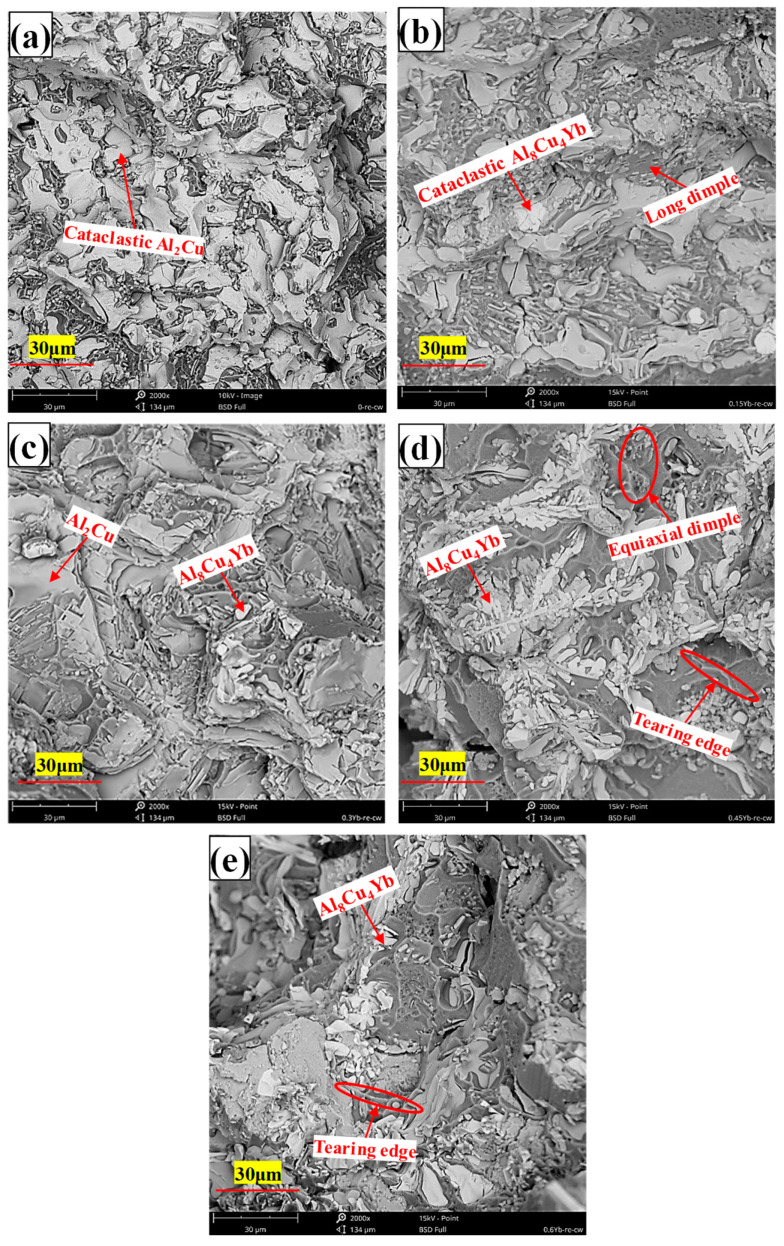
Tensile fracture morphology of T6 heat-treated alloy at room temperature ((**a**) = 0%Yb; (**b**) = 0.15%Yb; (**c**) = 0.3%Yb; (**d**) = 0.45%Yb; (**e**) = 0.6%Yb, wt.%).

**Figure 14:**
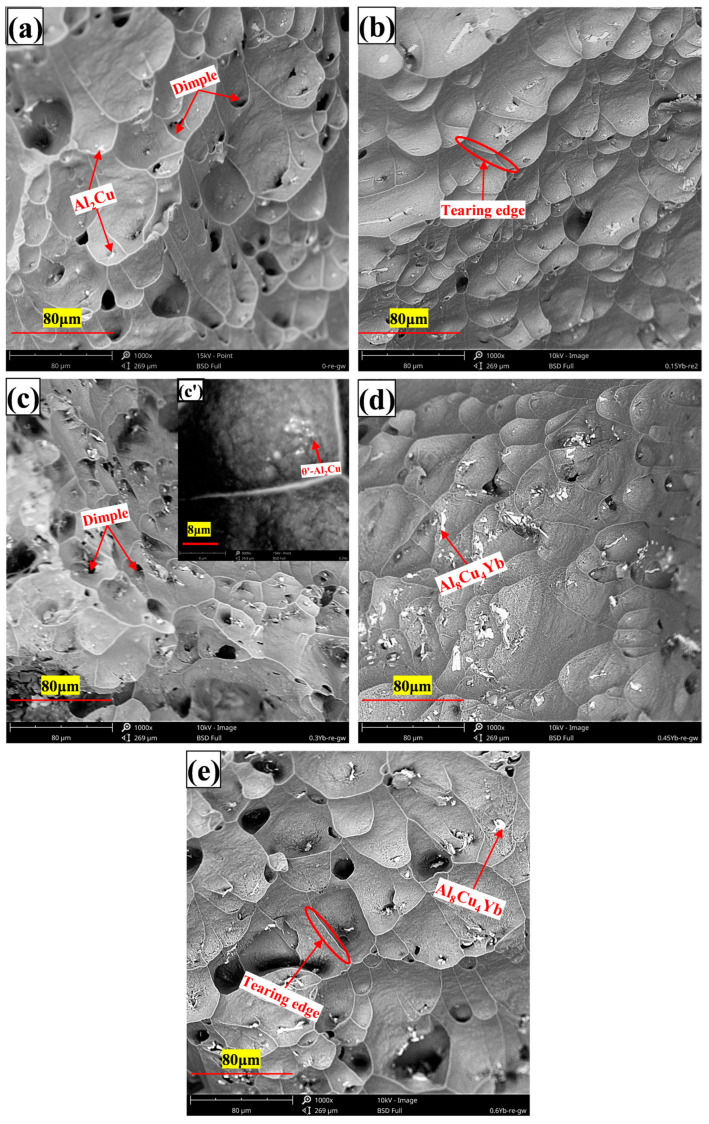
Tensile fracture morphology of T6 heat-treated alloy at high temperature ((**a**) = 0%Yb; (**b**) = 0.15%Yb; (**c**,**c’**) = 0.3%Yb; (**d**) = 0.45%Yb; (**e**) = 0.6%Yb, wt.%).

When the amount of Yb increases, more coarse and irregular Al_8_Cu_4_Yb phases are formed in the as-cast alloy matrix (see Figure 3). This phase belongs to the brittle phase of metal compounds with good thermal stability, so its size and quantity do not change much after T6 heat treatment, and the interface between this phase and the matrix becomes a stress concentration area. When Al_8_Cu_4_Yb is relatively thick, it will directly break from the phase, as shown in Figure 13b–e. When stretched at high temperatures, relatively large dimples will appear around the Al_8_Cu_4_Yb phase of large particles, as shown in Figure 14d,e, formed by the polymerization of micropores generated by stress concentration. Therefore, when Yb is added more, the tensile mechanical properties in the room and at high temperatures will decrease.

As can be seen from Figure 14, the tensile section at high temperatures is entirely made of dimples, indicating that the alloy is mainly ductile fracture at high temperatures, which can also be proved by the sizeable post-fracture elongation value of the alloy in Table 5. When we carefully observe the size of the dimple in the figure, it is found that the dimple size of the matrix alloy without Yb is more significant than that of the alloy with Yb. When the content of Yb is 0.3 wt.%, the alloy has the smallest fracture dimple size and the most dense distribution. Our analysis shows that, under normal circumstances, the dimple is formed by the polymerization and growth of micropores at the interface around the larger second-phase particles because the stress is often concentrated around the larger second-phase particles, coupled with the weak interface bonding. These areas are most likely to crack and form micropores first, and the micropores around the particles increase with matrix deformation. Finally, the micropores coalesce and connect to form a pit containing the particles, and the particles reside at the bottom of the center of the dimple. Due to the dense θ′-Al_2_Cu particles precipitated from the matrix after the addition of Yb, the dislocation slip and plastic deformation of the matrix are strongly hindered, so the tear of the dimple is shorter, the dimple size is small, and the depth is shallow, which is ultimately due to the inhibition effect of θ′-Al_2_Cu particles. The interface of the small dispersed nano-θ′-Al_2_Cu particles itself does not produce stress concentration, plus it has a coherent or semi-coherent interface with the matrix, and the interface is firmly combined. No micropores are generated around these tiny particles, so it does not produce its own dimple-shaped but is distributed on the inner wall of the dimple-shaped by large particles, as shown in Figure 14c’.

**Figure 15:**
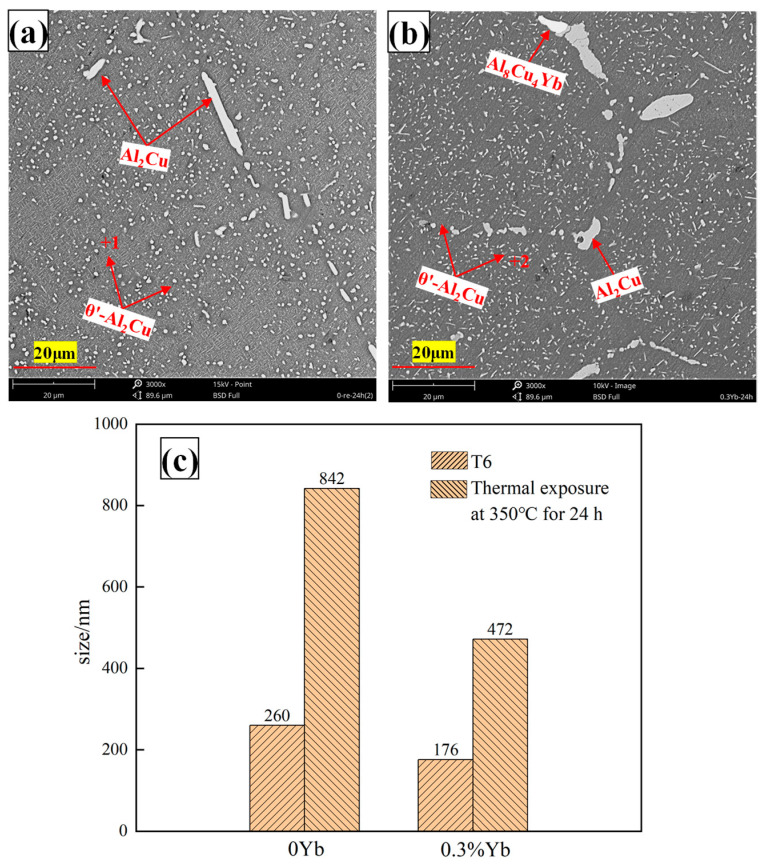
Microstructure and phase size changes of T6 heat-treated alloys after 24 h of heat exposure at 350 °C ((**a**) = 0%Yb; (**b**) = 0.3%Yb, wt.%; (**c**) dimensional changes of phases).

**Figure 16:**
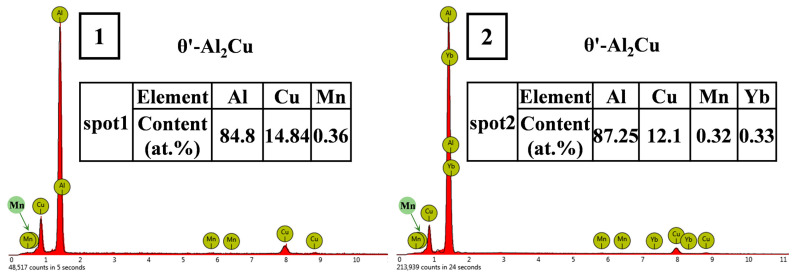
EDS analysis of points 1 and 2 in Figure 15.

For the same precipitated phase, the strength of the precipitation at room temperature is mainly determined by the number and size of the precipitated phase. The more precipitated phase, the smaller the size, and the more uniform the distribution, the more significant the strengthening effect. However, at high temperatures, the strength and thermal stability of the precipitated phase are also important factors affecting the strengthening effect. For this reason, 24 h heat exposure experiments were carried out on the heat-treated base alloy and the alloy with a Yb content of 0.3 wt.%. The microstructure is shown in Figure 15, and the analysis of the EDS points corresponding to points 1 and 2 in the figure is shown in Figure 16. It can be seen that the size of θ′-Al_2_Cu particles in both alloys increases after thermal exposure. Statistical analysis shows that the θ′-Al_2_Cu particle sizes of the matrix alloy and 0.3 wt.% Yb alloy coarsens by 224% and 168%, respectively, after 24 h of thermal exposure. The coarsening rate of θ′-Al_2_Cu particles in the base alloy without Yb is higher than that of the Yb-added alloy, which suggests that Yb also improves the thermal stability of the θ′-Al_2_Cu phase. This is also why adding Yb enhances the alloy’s high-temperature mechanical properties.

As we know, the atomic radius of Yb is 0.194 nm, the atomic radius of Al is 0.143 nm, and the atomic radius of Cu is 0.128 nm. The atomic radius of Yb is very different from that of Al and Cu, so the solubility of Yb in the aluminum matrix or Al_2_Cu is extremely low. Yb atoms do not easily replace Al and Cu atoms in aluminum matrix or θ′-Al_2_Cu. Yb atoms are more inclined to be segregated at the interface between Al_2_Cu and the matrix during the aging process. In addition, due to the difference of thermal diffusion coefficients of the elements in the matrix, in the aging process, Mn atoms with relatively high thermal diffusion coefficients may first arrive at the Al/θ′-Al_2_Cu semi-eutectic interface to control the coarsening of the interface in a reasonable range. Then, Yb atoms with relatively low thermal diffusion coefficients arrive at the interface. Finally, Mn and Yb co-segregate at the Al/θ′-Al_2_Cu semi-coherent interface, forming a new stacking mode, which can further reduce the interfacial energy or lattice mismatch, thus limiting the coarsening of θ′-Al_2_Cu and improving its thermal stability. Fuller et al. [52] found that the interfacial energy between the precipitated phase and the matrix and the diffusion rate between solute atoms are the two main factors affecting the coarsening of the precipitated phase. The reduction of the interfacial energy between the dispersed second-phase particles and the matrix can reduce the precipitated phase’s coarsening rate and improve the second phase’s thermal stability, improving the alloy’s mechanical properties at room and high temperatures [53].

## 4. Conclusions

This paper investigates the effect of Yb on the microstructure and room temperature and high-temperature mechanical properties of Al-6Cu-0.4Mn alloy in both the as-cast and T6 heat-treated states. The following conclusions can be drawn:

(1) The as-cast Al-6Cu-0.4Mn-xYb alloy mainly consists of α-Al, θ-Al_2_Cu, and Al_8_Cu_4_Yb phases. Yb can refine the α-Al grain and α-Al + θ-Al_2_Cu eutectic structure in the as-cast alloy. The refining effect of α-Al grain and eutectic structure increased at first and then decreased with the increase of Yb addition, and the refining effect was the best when the Yb addition was 0.3 wt.%. When the addition of Yb reaches 0.45 wt.%, the grain refinement effect begins to weaken, and the coarse Al_8_Cu_4_Yb phase will be precipitated. After T6 heat treatment, there is still a small amount of undissolved θ-Al_2_Cu phase in the matrix. Adding Yb can promote the precipitation and refinement of the θ′-Al_2_Cu phase when the content of Yb is 0.3 wt.%, θ′-Al_2_Cu particles have the smallest size and the largest number, and the average particle size is about 176 nm. The size and quantity of the Al_8_Cu_4_Yb phase have hardly changed after solution aging treatment, indicating the Al_8_Cu_4_Yb phase has good thermal stability up to 540 °C.
(2) The addition of the Yb element can improve the mechanical properties of as-cast and T6 heat-treated alloys at room temperature and 350 °C. The mechanical properties of as-cast and heat-treated alloys at room and high temperatures generally increased first and then decreased with Yb content. The ultimate tensile strength of the as-cast alloy containing 0.3 wt.%Yb is the highest at both room temperature and high temperature, which are 174.85 MPa and 95.46 MPa, respectively, which are 14.21% and 13.65% higher than that of the base alloy, respectively. The ultimate tensile strength of the heat-treated alloy containing 0.3 wt.%Yb also reached the highest at room temperature and high temperature, which were 328.98 MPa and 142.26 MPa, respectively, and increased by 21.63% and 17.71% compared with the base alloy, respectively.
(3) The main reason for improving mechanical properties at room temperature and high temperature of the as-cast alloy is that adding Yb promotes the refining of α-Al grains. A small amount of Al_8_Cu_4_Yb phase can also play a role in the second phase strengthening, and excessive coarse Al_8_Cu_4_Yb has adverse mechanical properties. The main reason for improving the mechanical properties of heat-treated alloys at room and high temperatures is that Yb promotes the precipitation and refinement of the θ′-Al_2_Cu phase and enhances the dispersion strengthening effect. However, Yb with a low thermal diffusion coefficient can promote the nucleation and precipitation of the θ′-Al_2_Cu phase through the “congestion” effect during aging. Heat exposure experiments show that Yb can improve the coarsening resistance of the θ′-Al_2_Cu phase at 350 °C. It is suggested that in the presence of Mn, the addition of Yb may induce co-segregation at the Al/θ′-Al_2_Cu semi-coherent interface to form a new interface stacking mode, which is conducive to reducing the interface energy, thus inhibiting the coarsening of θ′-Al_2_Cu and improving the thermal stability.

## Figures and Tables

**Table 1:** Nominal composition of Al-6Cu-0.4Mn-xYb alloy (wt.%).

Alloys Number	Al	Cu	Mn	Yb
1	Bal.	6	0.4	0.00
2	Bal.	6	0.4	0.15
3	Bal.	6	0.4	0.30
4	Bal.	6	0.4	0.45
5	Bal.	6	0.4	0.60

**Table 2:** Tensile properties of as-cast alloy at room temperature (27 °C).

Yb/wt.%	UTS/MPa	YS/MPa	FS/%	El/%
0	153.09 ± 7.0	96.19 ± 5.6	12.10 ± 2.1	4.69 ± 1.4
0.15	173.15 ± 9.3	104.97 ± 7.6	13.38 ± 3.4	5.78 ± 2.5
0.3	174.85 ± 10.5	113.84 ± 9.8	14.09 ± 4.0	6.35 ± 2.3
0.45	160.44 ± 10.1	103.70 ± 8.4	12.57 ± 2.6	4.88 ± 1.9
0.6	156.11 ± 8.4	93.76 ± 7.1	9.73 ± 1.9	3.18 ± 1.2

**Table 3:** High-temperature (350 °C) tensile properties of as-cast alloy.

Yb/wt.%	UTS/MPa	YS/MPa	FS/%	El/%
0	83.99 ± 4.5	71.54 ± 5.5	24.81 ± 4.2	20.22 ± 3.0
0.15	93.86 ± 6.9	82.85 ± 8.3	19.92 ± 3.8	16.91 ± 2.1
0.3	95.46 ± 8.4	85.21 ± 7.8	20.49 ± 3.8	17.52 ± 3.1
0.45	93.93 ± 7.9	82.99 ± 8.0	17.83 ± 2.9	15.24 ± 2.5
0.6	90.06 ± 5.1	78.65 ± 7.4	15.54 ± 2.5	11.21 ± 2.2

**Table 4:** Room temperature tensile properties of heat-treated alloy.

Yb/wt.%	UTS/MPa	YS/MPa	FS/%	El/%
0	270.48 ± 8.5	203.26 ± 8.4	8.82 ± 1.6	3.42 ± 1.2
0.15	302.29 ± 10.3	248.12 ± 9.5	11.94 ± 2.4	5.52 ± 2.3
0.3	328.98 ± 11.4	272.86 ± 10.9	11.65 ± 2.7	5.36 ± 2.4
0.45	300.18 ± 10.2	239.12 ± 11.4	11.34 ± 2.1	4.84 ± 2.2
0.6	289.06 ± 9.4	218.51 ± 10.3	10.74 ± 1.9	3.99 ± 1.8

**Table 5:** High-temperature tensile properties of T6 heat-treated alloy.

Yb/wt.%	UTS/MPa	YS/MPa	FS/%	El/%
0	120.86 ± 5.7	101.61 ± 6.1	18.67 ± 4.3	16.22 ± 3.0
0.15	131.76 ± 6.4	114.74 ± 7.4	16.67 ± 4.1	14.82 ± 3.2
0.3	142.26 ± 7.5	124.54 ± 8.6	15.25 ± 3.4	13.94 ± 2.8
0.45	137.07 ± 7.4	120.12 ± 9.1	13.14 ± 2.2	11.14 ± 2.4
0.6	123.26 ± 5.6	102.68 ± 5.0	12.08 ± 1.8	10.23 ± 1.9

**Table 6:** Comparison of tensile strength with other heat-resistant aluminum alloys.

Materials (wt.%)	Temperature (°C)	UTS (MPa)	Year	Ref.
Al-6Cu-0.4Mn-0.3Yb	350	142.3	2025	Present work
Al-5Cu-1.5Ni-0.3V	350	111.8	2024	[39]
Al-6Cu-0.4Mn-0.4Ag	350	135.9	2024	[25]
rGO/Al	350	128.0	2020	[40]
Al-12Si-4Cu-2Ni-1Mg-AlNp	350	106.0	2019	[41]
Al-6.5Cu-2Ni-0.5Zr-0.3Ti-0.25V	350	127.5	2023	[42]
Al-12.95Si-3.57Cu-0.72Mg-0.91Ni-0.53Fe-0.4Er	350	117.0	2019	[43]
Al-5Cu-0.2Mn-0.2Zr	300	124.9	2022	[44]
Al-7Si-Cu-0.5Mg-0.5Cr-0.4Ti-0.4V-0.25Zr	300	197.4	2016	[45]
Al-Cu-Mg-Ag-Sc	400	>100	2023	[46]
Al-7Si-2Cu-0.2Zr	200	246.0	2018	[47]
Al-7.38Si-0.36Mg-0.14Ti-0.22Er	200	220.6	2017	[48]

## Data Availability

The original contributions presented in this study are included in the article. Further inquiries can be directed to the corresponding author.
